# Molecular Dynamics‐Assisted Interaction Between HABT and PI3K Enzyme: Exploring Metastable States for Promising Cancer Diagnosis Applications

**DOI:** 10.1002/jcc.70080

**Published:** 2025-03-24

**Authors:** Rodrigo Mancini Santos, Teodorico Castro Ramalho

**Affiliations:** ^1^ Laboratory of Molecular Modelling, Department of Chemistry Federal University of Lavras Lavras Minas Gerais Brazil; ^2^ Centre for Basic and Applied Research, Faculty of Informatics and Management University of Hradec Králové Hradec Králové Czech Republic

**Keywords:** biased MD simulations, cancer, ESIPT, fluorescent sensors, spectroscopic probes

## Abstract

Local nonequilibrium approach has been used for many purposes when dealing with biological systems. Not only for unraveling important features of cancer development, a disease that affects the lives of many people worldwide, but also to understand drug–target interactions in a more real scenario, which can help to combat this disease. Therefore, aiming to contribute to new strategies against cancer, the present work used this approach to investigate the spectroscopy of 2‐(2′‐hydroxy‐4′‐aminophenyl)benzothiazole (HABT) when interacting with the PI3K enzyme, a widely associated target for the mentioned illness. The study consisted of evaluating the Excited State Intramolecular Proton Transfer (ESIPT) performance of HABT, in spectroscopic terms, when interacting with the PI3K enzyme in a local nonequilibrium regime. This scenario could be considered by investigating the metastable states of HABT in this system. From this, it was possible to observe that the ESIPT performance of HABT considerably differs when comparing the solution and protein environments, where 63% have appropriate geometry in the protein environment, against 97% in the aqueous environment. Thus, from an entirely theoretical methodology, the present work provides insights when modeling biological systems and contributes significantly to a better comprehension of promising probes for cancer diagnosis.

## Graphical abstract

Metastable states of the HABT molecule when docked into the PI3K protein drive its spectroscopic parameters. From biased MD simulations and PCA analysis, a new approach, considering a nonequilibrium perspective, was considered for signaling the PI3K protein, a crucial enzyme in cancer development, aiming for a promising cancer diagnosis application. A considerably different performance for the ESIPT process was observed when comparing the aqueous and protein environments.

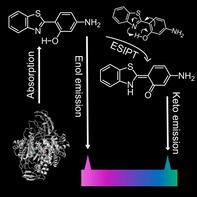

## 1 Introduction

In 1944, Schrödinger added a new perspective on how living systems work with his book *What is Life? The Physical Aspect of the Living Cell* [1]. Schrödinger's approach was quite innovative at that moment, and the main reason is that he considered living systems as open systems, which keep many processes in a local nonequilibrium regime [2, 3].

Local nonequilibrium states have recently become a valuable tool for modeling biological systems [3]. From this scenario, it was possible to investigate the fold and unfold events of proteins through membrane transport, cell signaling, and many other fundamental processes that occur in living systems [4, 5, 6]. In addition to that, the local nonequilibrium approach was used in many works to investigate ligand interaction with target enzymes, a crucial step in drug design and optimization [7, 8, 9].

Furthermore, one of the primary objectives of using a nonequilibrium perspective was to unravel the genetic and epigenetic causes of tumor development, which can lead to cancer [10, 11]. It is well known that cancer is one of the leading causes of death worldwide, being estimated that one in five people will develop cancer during their lifetime [12, 13]. Therefore, efforts to understand and combat this illness are of great value.

In this context, current cancer treatments are often insufficient to provide complete protection against the disease while also being prohibitively expensive, limiting accessibility [14, 15]. Moreover, the development of new treatments is highly complex, encountering significant knowledge barriers [16, 17]. An alternative strategy is to develop an effective diagnosis. In fact, an effective diagnosis considerably improves the survival rate of the patients affected by this disease, allowing intervention at an early stage, preventing the development and lethality of cancer [18].

With that in mind, in the pursuit of developing new compounds for cancer diagnosis, previous studies have highlighted a crucial aspect of drug development: drug–target interactions. It has been observed that a significant portion of drug–target interactions involving clinically approved small molecules is of a nonequilibrium nature [19, 20, 21, 22]. Therefore, this investigation provides highly valuable insights that could significantly enhance cancer diagnosis by incorporating the necessary complexity to address this challenge more effectively.

Aiming for a better, biocompatible, and effective diagnosis of cancer, fluorescent spectroscopic probes can be a great choice for this task [23, 24, 25, 26]. When compared to other spectroscopic probes, fluorescent sensors present high selectivity, low cost, and better biocompatibility [25, 26]. For this, many photophysical phenomena can explain their action, like the Excited State Intramolecular Proton Transfer (ESIPT) [25, 27].

Molecules capable of performing ESIPT, such as phenylbenzothiazoles (PBTs), can emit light at two different wavelengths [27]. In some cases, dual emission fluorescence can occur when the ESIPT process takes place [28]. It should be kept in mind that, however, a complete design of fluorescent probes often invokes an ESIPT on/off mechanism, where the most common is that, when signaling the analyte, the ESIPT is turned off, substantially increasing enol emission [29, 30].

In addition to that, it is important to mention that the ESIPT is often referred to as ultrafast, as it occurs on a femtosecond (10^−15^ s) scale [28, 31]. Therefore, considering the nonequilibrium nature of biological systems and the far‐from‐equilibrium time scale of the ESIPT process, it is crucial to evaluate the effects of metastable states of promising sensors in the ESIPT process. Furthermore, it can provide valuable insights into the on/off mechanisms of ESIPT in biological environments, offering important guidance for identifying new candidates for cancer diagnosis applications.

In that regard, promising fluorescent sensors capable of performing ESIPT could be used for signaling enzyme targets related to cancer development. In this sense, the Phosphoinositide 3‐kinase (PI3K) protein may be a great target, once it is widely associated with the progression of the mentioned illness [32, 33, 34, 35, 36, 37]. Now, considering a promising sensor for this target, the 2‐(2′‐hydroxy‐4′‐aminophenyl)benzothiazole (HABT) can be an appropriate probe, once it is capable of performing ESIPT, and there is evidence of strong interaction between similar structures and the PI3K protein [38, 39, 40, 41]. Therefore, evaluating HABT in signaling the PI3K target, adding the complexity of a local nonequilibrium regime, can be a powerful tool when aiming for valuable insights for an improvement of cancer diagnosis.

However, from a methodological point of view, the nontriviality of this task considerably hampers such a goal. Despite that, the work published by Ivernizzi and Parrinello [42] provided a fresh new methodology for accessing metastable states of molecular systems, where this could be done by enhancing the sampling of rare events in MD simulations.

From a novel perspective and methodology, this work aims to evaluate how the metastable states of HABT impact its spectroscopy when interacting with the PI3K protein target. Additionally, this investigation could offer valuable insights into the potential of HABT as a fluorescent probe for cancer diagnosis.

## 2 Computational Methods

### 2.1 HABT Structure and Parametrization

**FIGURE 1:**
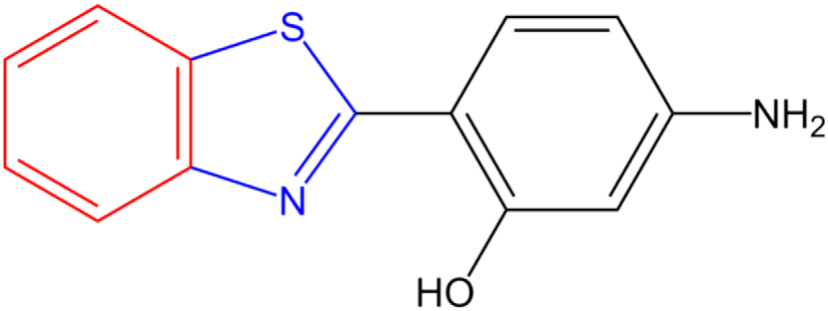
HABT structure, a benzothiazole derivative, constituted from a benzene (red), thiazole (blue), and phenyl (black).

The studied molecule was the 2‐(2′‐hydroxy‐4′‐aminophenyl)benzothiazole (HABT, Figure 1). For that, the HABT molecule's structure was constructed using GaussView 6 [43] software.

After that, it was necessary to optimize the structure and generate parameters for the force field. Thus, for structure optimization, Gaussian 09 software [44] was used, with B3LYP functional [45] and 6‐311 g basis set [46]. Therewith, the Automated Force Field Topology Builder (ATB) 3.0 [47, 48] was used for force field parameter obtention.

### 2.2 Molecular Docking

The Docking calculation was done using the optimized HABT structure and the PI3K crystallographic structure provided by D'Angelo et al. (PDB ID: 3QJZ) [49], as it also provides the active ligand docked in the active site of PI3K. As protein preparation, non‐polar hydrogens were added in order to consider the physiological pH (~7.4). Therefore, using the same strategy as Santos et al. [50], the position of the active ligand was used as a guide for the positioning of the HABT in the PI3K cavity.

The calculation was performed considering a simulation box (*x*, *y*, *z*) with a size (16 Å, 14 Å, 14 Å), where the coordinates are reported in Supporting Information S1. In total, 100 poses were simulated and had their energies calculated. For this, AutoDock Vina 1.1.2 software [51, 52] was used.

From the calculations, one orientation was selected as the optimal configuration and was used as the starting point for the molecular dynamics (MD) simulations [53]. The selection of the optimal orientation was based on the calculated energy and the presence of interactions previously reported for similar structures in the study of Santos et al. [50].

### 2.3 Unbiased MD Simulations

With the HABT structure and force field parameters, unbiased MD simulations were carried out. In this scenario, three MD simulations of 100 ns were carried out, one for each studied system: HABT in vacuum (1), HABT in water (2), and protein‐docked HABT in water (3). In order to simplify the text, the systems were named as HABT (1), HABT+ WAT (2), and HABT + WAT + PROT (3).

The simulations were carried out using GROMACS 2021.4 software [54]. For that, the GROMOS 54a7 force field [55] was used both for the protein and ligand. For water, the simple point charge (SPC) model was used. The performed simulations were done at a temperature of 300 K, with a timestep of 2 fs. The simulations consisted of the steps of minimization, NVT equilibration, NPT equilibration, and production (see Supporting Information S1 for details).

### 2.4 Biased MD Simulations

Three biased MD simulations of 100 ns were carried out, each for each of the three studied systems, using the same setup as the unbiased ones. From the biased trajectory, it was possible to access different metastates. The technique used for such a goal was the on‐the‐fly probability enhanced sampling (OPES) expanded, developed by Ivernizzi et al. [56].

The biased MD simulations were carried out by biasing the potential energy *U*, using as a target the multicanonical ensemble over a temperature range of 300–600 K, with a pace of 500 (1 ps). Biasing the potential energy, which is possible for the multicanonical approach, can be a good strategy when there is no previous knowledge of the system, which hampers the identification of good CVs [56].

In order to perform the biased MD simulations, PLUMED 2 v2.8.0 software [57] patched with GROMACS 2021.4 was used, and the GROMOS 54a7 force field was also used.

### 2.5 Principal Component Analysis (PCA) and Conformational Selection

After the calculation of descriptors from biased MD trajectories (shown in Section 3), PCA analysis was carried out for all three studied systems. From PCA analysis, it was possible to analyze how the accessed metastable states were distributed and then characterize them in terms of the chosen descriptors. For PCA analysis, the python library scikit‐learn (version 1.2.2) [58] was used.

Therefore, after identification and characterization of the accessed metastable states, representative conformations were selected from each metastate of system 3. For that, the mean value of the descriptors was calculated in each identified metastate. After that, in the unbiased MD trajectories, the conformations whose descriptor values were closest to the calculated averages of their respective metastate were selected for quantum calculations.

### 2.6 QM Calculations Setup

QM calculations were carried out using the selected conformations. With the selected HABT structure from the unbiased trajectory, residues were also collected to simulate the protein environment in QM calculations. The residue selection was based on an h‐bond and root mean square deviation (RMSD) analysis conducted for system 3.

In this scenario, single‐point DFT and TDDFT calculations were carried out using Gaussian 09 software [44] for vertical excitation energy investigation. The calculations were performed to construct the following path: enol configuration at ground state (1), enol configuration at the first excited state (2), keto configuration at the first excited state (3), and keto configuration at ground state (4). The single‐point calculations were performed with the optimized geometries in each state mentioned above, where only the HABT geometry was optimized, and the amino acid residues were maintained fixed.

For both optimization and single‐point calculations in the ground state, the B3LYP functional [45] was used with the tzvp basis set [59]. Now, for the excited states optimization and single‐point calculations, the CAM‐B3LYP functional [60] was used with the same basis set. Both B3LYP and CAM‐B3LYP functionals are widely used to study benzothiazole derivatives, where the tzvp basis set follows the same [61, 62, 63].

## 3 Results and Discussion

### 3.1 Docking

**FIGURE 2:**
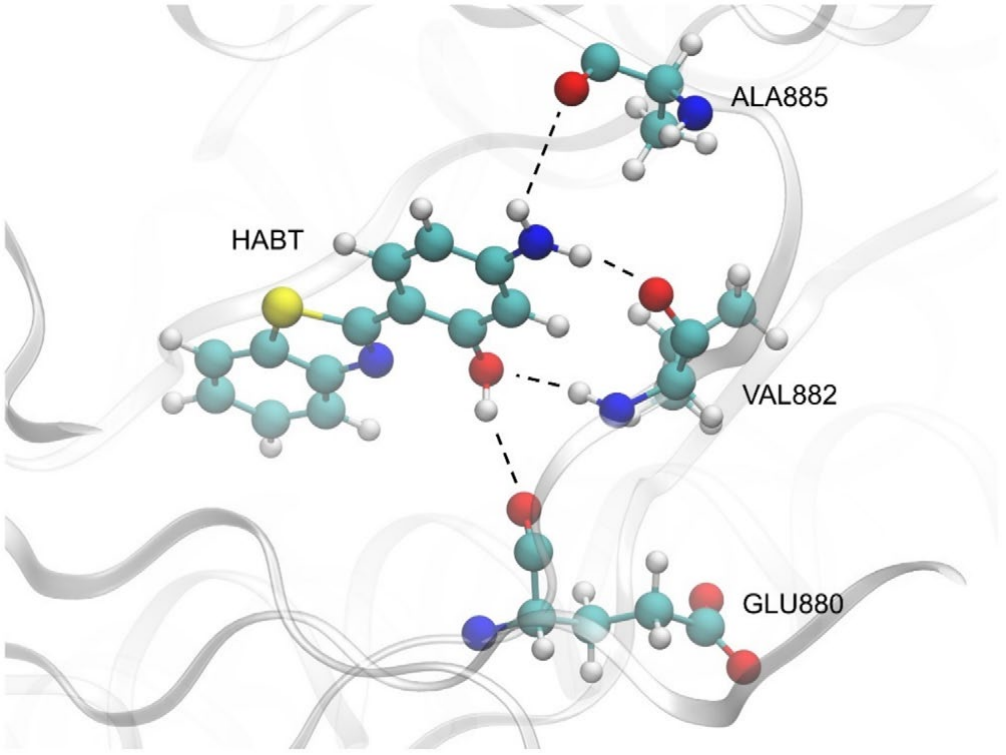
Best orientation obtained from Docking calculations. At the bottom right, GLU880 forms one h‐bond interaction (2.51 Å) with HABT. At the middle right, VAL882 forms two h‐bond interactions (2.20 and 2.33 Å) with HABT. At the top right, ALA885 forms one h‐bond interaction (2.98 Å) with HABT.

The Docking calculation analyzed 100 poses, where the best one is shown in Figure 2. As shown, four h‐bond interactions were observed between the HABT and the amino acid residues glutamic acid (GLU880), valine (VAL882), and alanine (ALA885). This orientation showed a favorable interaction with the PI3K cavity, with an energy of −7.3 kcal/mol.

In Figure 2, it was possible to notice that HABT can act as a donor and an acceptor of electrons. In the interaction with the glutamic acid residue (2.51 Å), the HABT acts as an electron donor (HABT:H18···O:GLU880). Now, by observing the two interactions with the valine residue, in the first one (2.20 Å), the HABT acts as an electron acceptor (HABT:O···HN:VAL882), and in the second one (2.33 Å), as an electron donor (HABT:NH1···O:VAL882). In its last interaction, which occurs with the alanine residue (2.98 Å), the HABT acts as an electron donor (HABT:NH2···O:ALA885).

In a previous study, Santos et al. [50] showed similar interactions when performing docking calculations for cis‐dichloro(2‐aminomethylpyridine) platinum(II) bonded to 2‐(4′‐amino2′‐hydroxyphenyl)benzothiazole (AHBT) in the same PI3K cavity. Due to the similarity of the structure of AHBT and the bonded structure of the platinum complex, the same h‐bond interactions between AHBT and VAL882, as well as AHBT and GLU880, were observed in the present docking study. However, in the current scenario, since there is no platinum group at the amino edge of AHBT, a new h‐bond interaction could be observed between AHBT and ALA885.

### 3.2 Unbiased MD Simulation Analysis

As a starting point, unbiased MD simulations were carried out for all three studied systems: HABT (1), HABT+WAT (2), and HABT+WAT + PROT (3). Understanding the simulation environment is crucial for obtaining insights for further metastable state characterization. Hence, an RMSD analysis was performed to evaluate the simulation stability and behavior for all studied systems.

Therefore, Figure S1 shows the obtained RMSD values for HABT in systems 1 and 2. For system 1, a mean RMSD value of 0.13 Å was found, and a standard deviation of 0.05 Å. Now, for system 2, a mean RMSD value of 0.15 Å was obtained, and a standard deviation of 0.05 Å. The values found for both systems imply simulation stability. Details about the interactions responsible for such an RMSD profile are in Supporting Information S1: Section SI.2.

**FIGURE 3:**
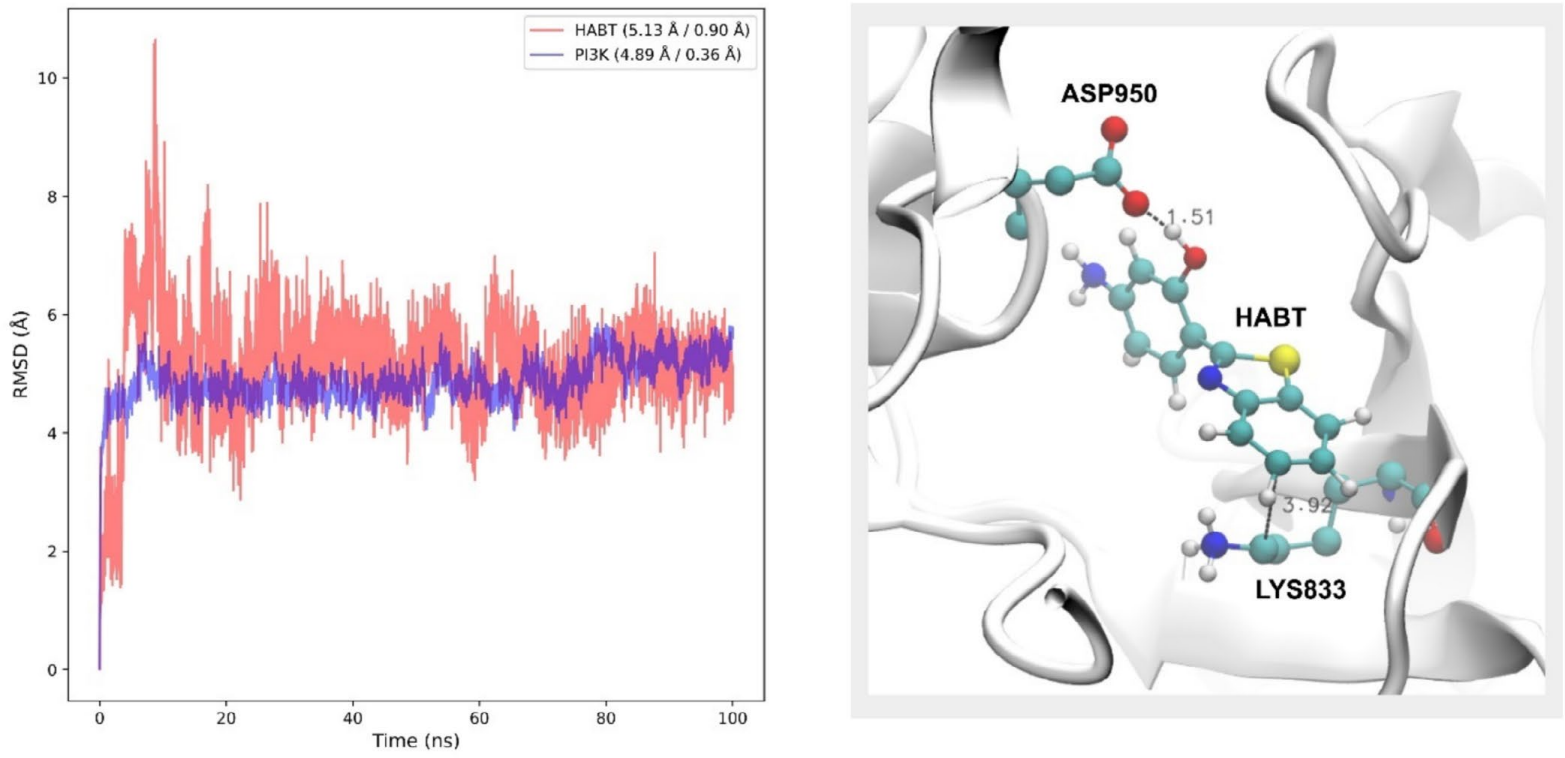
(a) RMSD (Å) vs. Time (ns), where the RMSD profile of HABT is shown in red and PI3K in blue. In the figure caption, the values of average/standard deviation appear. (b) Captured interactions between HABT and protein PI3K. The ASP950–HABT interaction occurs between the carboxilic group of ASP950 and the –OH group of HABT. The LYS833–HABT interaction occurs between the LYS833 carbonic chain and the benzene group of HABT.

Now for system 3, Figure 3a shows the obtained RMSD values of HABT and PI3K for each simulation step. In that regard, an RMSD average of 5.13 Å was obtained for the HABT molecule, with a standard deviation of 0.90 Å. For PI3K, an RMSD average of 4.89 Å was obtained, with a standard deviation of 0.36 Å. Therefore, the obtained values are considered low for large systems, implying reasonable stability for the performed simulation.

In addition, the obtained value for PI3K was close to the one obtained by Santos et al. [50], which was an RMSD mean of 3.68 Å and a standard deviation of 0.65 Å. Even though the mentioned published work used a different force field, the closer values reinforce the simulation stability.

For a better understanding of the nature of system 3, it is important to evaluate interactions that may help stabilize the observed evolution in Figure 3a. Therefore, an h‐bond analysis between the HABT and PI3K was performed using the Visual Molecular Dynamics (VMD) [64]. A cutoff radius of 3.0 Å and a cutoff angle of 30° were considered. In that regard, Figure 3b shows the chemical environment where interactions between HABT and the PI3K protein stabilize the observed evolution.

For system 3, much more relevant intermolecular h‐bonds can occur, since the HABT molecule was docked in the PI3K protein in this system. Therefore, from h‐bond analysis, it was found that the HABT:OH···O:ASP950 (aspartic acid residue) intermolecular h‐bond interaction (Figure 3b) represented 69.75% of these interactions. This interaction starts occurring after 20 ns of simulation production.

Meanwhile, at the beginning of the simulation, it was verified that h‐bonds with the valine residue (VAL882) represented 11.46% of the h‐bond interactions. However, this interaction did not hold with the simulation's evolution. Its occurrence was only observed before 20 ns of simulation, indicated by an RMSD instability until the mentioned simulation time.

Another important interaction, which is not captured in the h‐bond analysis, is the interaction between the lysine residue (LYS833) and the benzene portion of HABT (also shown in Figure 3b). It is important to mention that, despite this hydrophobic interaction not being as stabilizing as the mentioned h‐bond interactions, it follows from the simulation point that the HABT:OH···O:ASP950 h‐bond interaction starts occurring.

In this regard, after exploring interactions between the HABT molecule and its environment, valuable insights could be obtained, helping to understand the stabilization of the accessed metastable states.

### 3.3 Biased MD Simulations: Assessing Metastate Distribution and Evaluating the Solvent Effect

From biased trajectories, it was possible to evaluate the accessed metastable states for all three systems. For that, PCA analysis was conducted to identify and characterize the accessed metastable states. The characterization was made in terms of descriptors, specifically chosen considering the nature of the HABT molecule.

**FIGURE 4:**
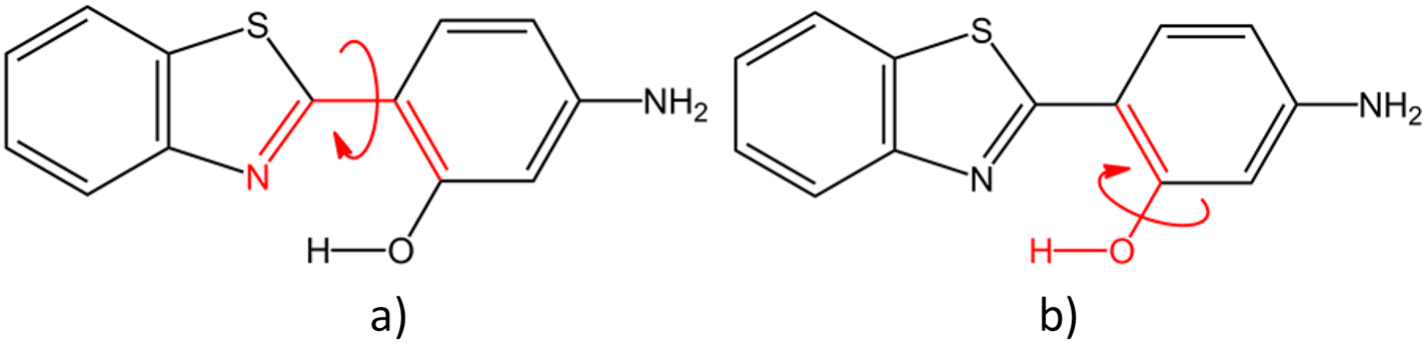
Chosen descriptors for PCA analysis are (a) dihedral 1 and (b) dihedral 2.

The descriptor's source is the obtained trajectory files ${\left(R\right)}_{i=1}^{N}$ of the biased MD simulations, where *i* goes up to the *N* steps of the performed simulation, and R represents cartesian coordinates. From that, dihedral angles (Figure 4) could be calculated and used as descriptors. The use of dihedral angles is appropriate once internal coordinates can provide a correct separation between the internal and overall motion [65].

**FIGURE 5:**
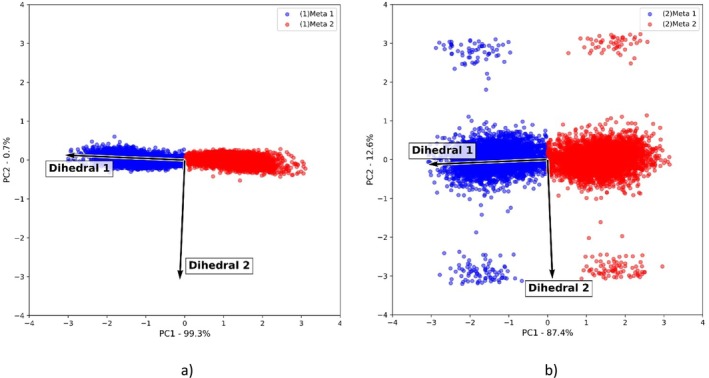
Biplot graph obtained after PCA analysis of (a) system 1 and (b) system 2.

With the calculated descriptors, it was possible then to perform PCA for all three systems. Hence, comparing the obtained metastates distribution of systems 1 and 2, it was possible to evaluate the solvent effect. For system 1 (Figure 5a), two long‐lived metastable states were accessed ((1)Meta 1 and (1)Meta 2), being differentiated by PC1.

After performing a population analysis, it was observed that (1)Meta 1 and (1)Meta 2 contain almost the same population, with 5012 conformations for (1)Meta 1 and 4989 conformations for (1)Meta 2. In this scenario, these conformations were differentiated only by a dihedral 1 (Figure 4a) fluctuation, presenting no considerable fluctuation of dihedral 2 (Figure 4b).

Now, for system 2 (Figure 5b), different from what was observed for system 1, after the solvation, it was possible to notice the appearance of new metastable states with a smaller population, differentiated by PC2. However, the two long‐lived metastable states were still observed for system 2, located around PC2 = 0.

After population analysis, it was shown that the newly appeared metastates presented a population that varies between 50 and 83 conformations. These newly accessed metastates are characterized by their dihedral 2 fluctuation, which has no considerable fluctuation for the main group (located around PC2 = 0). Therefore, the main group maintained almost the same population as observed for system 1, consisting of 4849 conformations for (2)Meta 1 and 4876 conformations for (2)Meta 2. Hence, after the addition of water, a relevant dihedral 2 fluctuation could be stabilized.

The presented results could be explained in terms of the possible interactions between HABT‐HABT and HABT‐environment. In this scenario, since system 1 was simulated in a vacuum, only intramolecular interactions can stabilize the accessed metastable conformations. Therefore, one important interaction that prevents dihedral 2 fluctuation is the –OH and –N= intramolecular h‐bond interaction (Figure S1b). Thus, once there is no other interaction capable of stabilizing the dihedral 2 fluctuation, the –OH and –N= groups are kept close enough to maintain this interaction, and no relevant dihedral 2 fluctuation is observed for system 1.

On the other hand, system 2 consists of HABT solvated in water. Hence, it was verified that, for system 2, relevant dihedral 2 fluctuations are stabilized by intermolecular hydrogen bonds between the hydrogen of the –OH group of HABT and the oxygen of water molecules (Figure S1d). However, the population analysis implies that the occurrence of this interaction is much lower than the –OH and –N intramolecular hydrogen bond interaction. In this sense, it was possible to conclude that only water is not capable of effectively stabilizing a relevant dihedral 2 fluctuation in long‐lived metastable states.

Therefore, by evaluating the solvent effect, valuable insights could be obtained about the descriptor's behavior. The discussed analysis showed that by adding possible intermolecular interactions to the system, dihedral 2 starts to gain relevance in explaining the metastates distribution of the HABT molecule. This is important since this knowledge led to a better comprehension of the obtained distribution for system 3.

### 3.4 Biased MD Simulations: Assessing the Distribution of Metastable States and Evaluating the Effect of the Protein Environment

**FIGURE 6:**
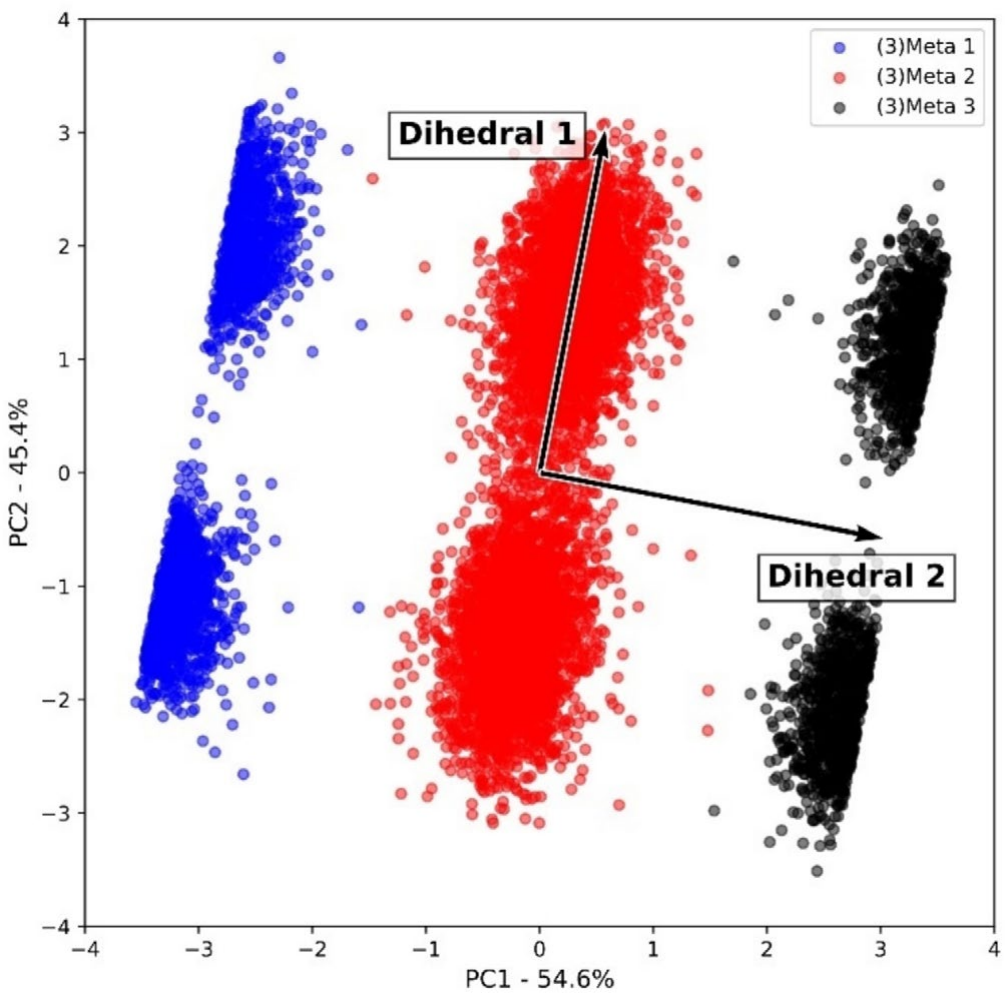
Biplot graph obtained after PCA analysis for system 3.

For system 3, a separate discussion was held, since this is the system of interest in this work, which will provide conformations for further ESIPT study with QM calculations. Differently from what was observed for systems 1 and 2, for system 3, three long‐lived metastates were accessed, separated in the PC1 axis, which contains 54.6% of the explained variance (Figure 6). Also, there was a substantial increase in the variance explained by PC2, which for system 3 is 45.4%, almost four times higher than the one observed for system 2.

In this scenario, population analysis showed that (3)Meta 2 contains almost 63% of the explored conformations, where 6294 conformations belong to (3)Meta 2. For those metastates at the ends of the PC1 axis ((3)Meta 1 and 3), approximately 37% belong to them, where (3)Meta 1 contains 1916 conformations and (3)Meta 3 contains 1791 conformations.

Furthermore, all three long‐lived metastates are decomposed into two shorter‐lived metastates, which are observed in the separation performed by the PC2 axis. This decomposition occurs in a way that the long‐lived metastate is split in half, where both clusters separated by the PC2 axis represent approximately 50% of the conformations of their respective long‐lived metastate.

Concerning the descriptor's behavior in the differentiation of the long‐ and shorter‐lived metastates, an inversion of relevance was observed when compared to systems 1 and 2. For system 3, dihedral 2 was relevant to differentiate long‐lived metastates. Now, dihedral 1 was relevant to differentiate the decomposition of the three long‐lived metastates; in other words, to differentiate the shorter‐lived metastates. Therefore, after docking the HABT molecule in the protein PI3K, it was observed a considerable increase in dihedral 2 fluctuation.

**FIGURE 7:**
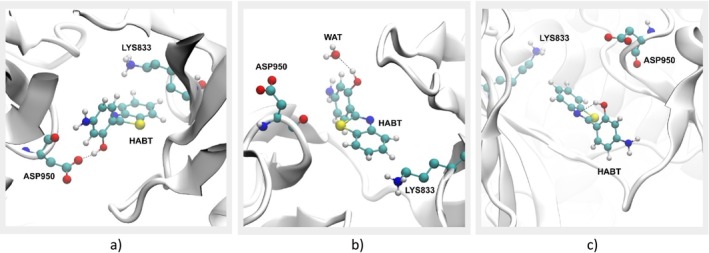
Molecular interactions that (a) stabilizes conformations of (3)Meta 1A and 3A, (b) stabilizes conformations of (3)Meta 1B and 3B, and (c) stabilizes conformations of (3)Meta 2A and 2B.

In Figure 7, it is possible to observe the interactions responsible for such observation. Despite intermolecular interactions between HABT and water helping stabilize the fluctuations, the main reason for an increase in the dihedral 2 fluctuation remains the new intermolecular interactions of HABT and amino acid residues of the protein PI3K.

Such interactions were partially explored in Section 3.2; however, the main goal here was to find specific interactions that stabilize each accessed metastable state of HABT in system 3. Therefore, an investigation was performed based on the previously performed h‐bond analysis. In order to clearly explain the observed interactions, the accessed shorter‐lived metastates were divided into (3)Meta *n*A and *n*B, where *n* is the number of the respective long‐lived metastate, and the labels A and B are associated with their locations in PC2. Figure S5 shows how the labeling was defined.

In Figure 7a, it was possible to observe that the conformations of (3)Meta 1A and 3A stabilize their dihedral 2 fluctuation by making an intermolecular h‐bond interaction between the –OH group of HABT and the carboxylic group of the ASP residue. Now, in Figure 7b, it was possible to observe that the conformations of (3)Meta 1B and 3B stabilize their dihedral 2 fluctuations by making an intermolecular h‐bond interaction between the –OH group of HABT and water molecules. This difference occurs once in the metastates 1B and 3B; the dihedral 1 fluctuation prevents the h‐bond interaction with the ASP residue. Finally, Figure 7c shows that the conformations of (3)Meta 2A and 2B are stabilized by the –OH and –N intramolecular h‐bond interaction. It is important to mention that, in all accessed metastable states, the LYS–HABT hydrophobic interaction is present, helping the stabilization of the dihedral 1 fluctuation.

Therefore, from the population analysis done in this subsection, it was possible to observe that the –OH and –N= intramolecular h‐bond interaction is the most stabilizing. This can be concluded in a scenario where almost 63% of the accessed conformations belong to (3)Meta 2. On the other hand, it is possible to observe that the protein environment was capable of stabilizing many more conformations than only the water environment. From systems 2 to 3, the conformations stabilized by h‐bond intermolecular interactions increased substantially. In that way, the results presented so far show the challenge when dealing with biological environments and the need for new approaches, like considering a nonequilibrium environment.

From our findings, the unbiased MD production confirmed that the equilibrium conformation (see Table S2), which is the most populated, has a geometry with a dihedral 1 of approximately 0°. However, the biased MD simulation results, which show geometries with dihedral 1 values different from 0°, are expected after applying enhanced sampling techniques, as these accessed conformations represent out‐of‐equilibrium states (for details, see Supporting Information S1: Section SI.7).

Despite the biased MD simulations providing valuable information about metastate distribution and stabilization, they do not provide information about ESIPT spectroscopy. For that, more accurate calculations, like QM calculations, should be performed. However, since QM calculations are much more computationally expensive, for a feasible analysis it is necessary to reduce the number of conformations that will go through expensive calculations. Therefore, selecting representative conformations of each accessed metastate may be a good strategy for the ESIPT study.

### 3.5 Fluorescence Properties in the Protein Environment

Representative conformations were selected and submitted to QM calculations, where it was possible to evaluate the ESIPT process for all selected metastable conformations. The selection procedure and the selected conformations are detailed in Supporting Information S1: Section SI.5.

**FIGURE 8:**
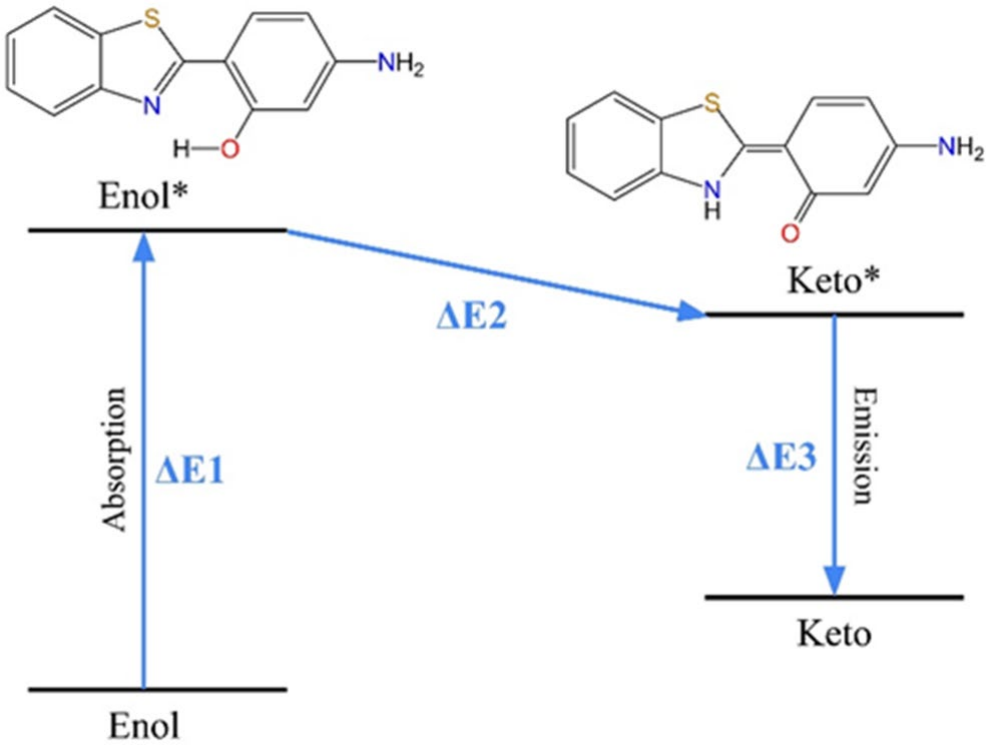
ESIPT scheme, where *ΔE*1, *ΔE*2, and *ΔE*3 represent the energy variation between the states, values which are presented in Table 1.

Therefore, to understand the obtained results, the collected data were summarized in Table 1, where the values contained in the table follow the values shown in Figure 8, being the energy difference between the states.

**TABLE 1:** Energy differences between states, in eV, according to that shown in Figure 8.

Conformations	*ΔE*1	*ΔE*2	*ΔE*3
1A	3.98	—	—
1B	3.84	—	—
2A	3.17	−0.03	2.28
2B	3.81	−0.50	2.68
3A	3.56	—	—
3B	3.70	—	—
EQ	3.37	−0.43	2.43

In this scenario, it was verified that the equilibrium conformation can perform ESIPT, thus following the expected [61]. However, when observing the obtained results, only the conformations belonging to (3)Meta 2 were capable of performing ESIPT, indicating only enol emission for those belonging to (3)Meta 1 and 3.

Therefore, what was possible to conclude in Tables 1 and S2 is that the ESIPT only occurs when there exists an intramolecular interaction by h‐bond between N···HO (Figure S1b). This conclusion is in accordance with the proposed mechanism for this reaction by Eigen and Weller [66, 67], as it reinforces the need for the h‐bond contact between the photoacid and the near basis. In that way, it was only verified that the occurrence of ESIPT is for the conformations where the distance N···HO is below 3.00 Å.

Hence, conformations 1A, 1B, 3A, and 3B did not present ESIPT occurrence. On the other hand, even though dihedral 1 fluctuation had a minor impact on favoring or disfavoring the ESIPT, its fluctuation did not prevent the ESIPT occurrence, since it did not inhibit the mentioned intramolecular interaction.

Therefore, at closer distances of intramolecular interaction by h‐bond between N···HO, the ESIPT process is favored. Of the three conformations that verified the ESIPT occurrence, being the conformations EQ, 2A, and 2B, the worst favored was the conformation 2A, with a *ΔE*2 of −0.03 eV. For this conformation, the distance N···HO was the highest, being 2.94 Å. Now for conformations 2B and EQ, it was verified to have a close value of *ΔE*2, being −0.50 eV for 2B and −0.43 eV for EQ. The distance N···HO values were lower than the one for 2A, being 2.61 Å for 2B and 2.30 Å for EQ.

Observing the spectroscopy of the conformations in which there is the occurrence of ESIPT, a near violet enol emission for all those conformations, *ΔE*1 of 3.17–3.81 eV, was observed. For conformations 2B and EQ, the enol emissions occur in the UVA region, being 325.42 and 367.91 nm, respectively. However, for conformation 2A, a higher wavelength of 391.12 nm, located in the visible violet, was observed.

For the keto emissions, for conformations 2A and EQ, a near green emission (ΔE3 of 2.28–2.43 eV) was observed, with 543.79 and 510.22 nm, respectively, while for the 2B conformation, a blue emission (ΔE3 of 2.68 eV), with 462.63 nm. Therefore, conformations 2A and EQ presented the highest stokes shift, with 152.67 and 142.31 nm, respectively, while conformation 2B presented a lower stokes shift, with 137.21 nm. Thus, all conformations where it was verified that the occurrence of ESIPT presented a considerable stokes shift, appropriate for promising fluorescent sensors. It is important to mention that the conformations capable of performing ESIPT represent 63% of all accessed conformations.

Now, for conformations of (3)Meta 1 and 3, competitive intermolecular h‐bond interactions caused an inappropriate geometry for ESIPT occurrence, verified by the fact that no *ΔE*3 emission was observed for those conformations. The inappropriate geometry could also be verified from the higher values of N···HO distance (Table S1), where these values are kept around 4.50 Å for those conformations. This increase is caused by dihedral 2 fluctuations, which are stabilized by h‐bonds with the surroundings. Conformations with geometries unsuitable for ESIPT performance account for 37% of all observed conformations.

The presented results enable a statistical comparison of performance between the solution‐only environment (system 2) and the protein environment (system 3). In system 2, 97% of the conformations exhibit geometries suitable for ESIPT, compared to only 63% in system 3. The proportion of conformations with ESIPT activated is significantly lower in system 3. These findings suggest that HABT exhibits a promising capability for enhanced enol emission when interacting with the PI3K protein, which could be advantageous for signaling applications [29].

## 4 Conclusions

This work provides valuable insights into the potential use of HABT for cancer diagnosis. In the protein environment, 63% of the observed conformations exhibit geometries suitable for ESIPT, while 37% do not. In contrast, in solution, the proportion of conformations capable of performing ESIPT increases significantly to 97%. These findings suggest that HABT is likely to exhibit enhanced enol emission when interacting with the PI3K protein, highlighting its potential as a promising fluorescent probe for cancer diagnosis.

In this scenario, dihedral fluctuations had a considerable impact on the ESIPT occurrence. Both dihedral 1 and 2 fluctuations had an impact on the HABT performance in ESIPT, where the latter was the most important. It was observed that fluctuations of dihedral 2 were crucial in preventing the ESIPT occurrence. That occurred in a scenario where these fluctuations were the cause of an inappropriate geometry for ESIPT occurrence. From this, the intramolecular N···HO h‐bond interaction could be inhibited, an interaction that is of great relevance for the tautomerization process.

In this sense, the present work could contribute as a step forward in the improvement of cancer diagnosis. From Schrödinger's point of view, explored in several works, it was possible to add a more realistic comprehension of biological systems necessary to investigate promising new probes for disease diagnosis.

Therefore, when working with theoretical methodologies, incorporating the necessary complexity into models is both the primary objective and a significant knowledge barrier. In this regard, the present study provides important methodological advancements and valuable mechanistic insights into metastable states. It offers a general approach to addressing ligand flexibility and metastable states within the protein active site, supported by reliable statistical information. However, it is important to note that the authors are aware of the limitations regarding the absolute values of spectroscopic emission, which should be interpreted with caution. In this context, our work encourages further research in the field to deepen the understanding of HABT's role as a fluorescent sensor for cancer diagnosis.

Thus, the present work shows that from an entirely theoretical methodology, it was possible to achieve fresh conclusions about the ESIPT process in biological environments, offering important guidance for identifying new candidates for use in cancer diagnosis applications.

## Supporting information


**Data S1.** Supporting Information.

## Data Availability

The data that support the findings of this study are available on request from the corresponding author. The data are not publicly available due to privacy or ethical restrictions.
