# Supplementary material for: Molecular Dynamics‐Assisted Interaction Between HABT and PI3K Enzyme: Exploring Metastable States for Promising Cancer Diagnosis Applications
Source: J Comput Chem. 2025 Mar 24;46(8):e70080. doi: 10.1002/jcc.70080 (PMC11933734; doi:10.1002/jcc.70080)
Supplement: Supplementary file 1 — Data S1. Supporting Information. [file JCC-46-0-s001.docx]

**SUPPORTING INFORMATION**

Molecular Dynamics-assisted interaction between HABT and PI3K enzyme: Exploring Metastable States for Promising Cancer diagnosis Applications

Rodrigo Mancini Santos,^[[1]](#endnote-1)^ Teodorico Castro Ramalho^i,^^[[2]](#endnote-2)^

Rodrigo Mancini Santos (E-mail: *rodrigomancini4@gmail.*com);

Teodorico Castro Ramalho (E-mail: [*teo@ufla.br*](mailto:teo@ufla.br)).

1. ^i^ Laboratory of Molecular Modelling, Department of Chemistry, Federal University of Lavras, Lavras 37200-000, MG, Brazil [↑](#endnote-ref-1)
2. ^ii^ Department of Chemistry, Faculty of Science, University of Hradec Králové, 500 03 Hradec Králové, Czech Republic

   **SI.1.1 Docking box coordinates**

   The constructed box coordinates for docking calculation are: center_x = 19.517, center_y = 13.916, center_z = 21.614; size_x = 16.00, size_y = 14.00, size_z = 14.00.

   **SI.1.2 Pre-MD production setup**

   Molecular dynamics were carried out for all three systems with 100 ns long, being them: HABT in vacuum (1), HABT in water (2), and PI3K-docked HABT in water (3). The simulations followed the steps of energy minimization, NVT equilibration and NPT equilibration before the production step.

   Therefore, for energy minimization, a timestep of 5 fs was used with steepest descent integrator. Energy minimization followed until maximum force converged to values lower than 10 kJ/mol. Now, for NVT equilibration, a dynamical timestep was used. In this sense, the NVT equilibration consisted of three steps, all of them using leap-frog integrator and velocity rescaling thermostat for temperature coupling, where the first step used a timestep of 0.5 fs during 10 ps, the second step used a timestep of 1 fs during 40 ps, and the third step used a timestep of 2 fs during 50 ps. Moving further, for the NPT equilibration a timestep of 2 fs was used with a total time of 100 ps, where Berendsen for pressure coupling, and velocity rescaling thermostat for temperature coupling was used.

   **SI.1.3 Unbiased MD production setup**

   Unbiased MD simulations production was carried out with the outputs of the NPT equilibration, where a timestep of 2 fs was used, being carried out for 100 ns long. For the production, Parrinello-Rahman with isotropic pressure coupling and a compressibility of 4.5 x 10^-5^ was used, and velocity rescaling thermostat for temperature coupling were used. In addition, for neighbor searching and Van der Waals interactions the Verlet cutoff scheme was used, with a cutoff of 12 Å, and Particle Mesh Edwald (PME) was used for long range electrostatic interactions, also with a cutoff of 12 Å. For this step, leap-frog integrator was used, and the simulation was performed in a temperature of 300K.

   **SI.1.4 Biased MD production setup**

   Biased MD simulations production was also carried out with the outputs of the NPT equilibration. For the production, the setup was the same as the one used for Unbiased MD productions. Therefore, the only inputs needed for the OPES expanded simulation was the temperature range, which was set to go from 300K to 600K, and the pace of bias potential update, which was set as 500 simulations steps (1 ps). In this scenario, the multicanonical target was reached by biasing the potential energy *U*.

   **SI.2 Unbiased MD analysis**

   **SI.2.1 Solvent Effect**


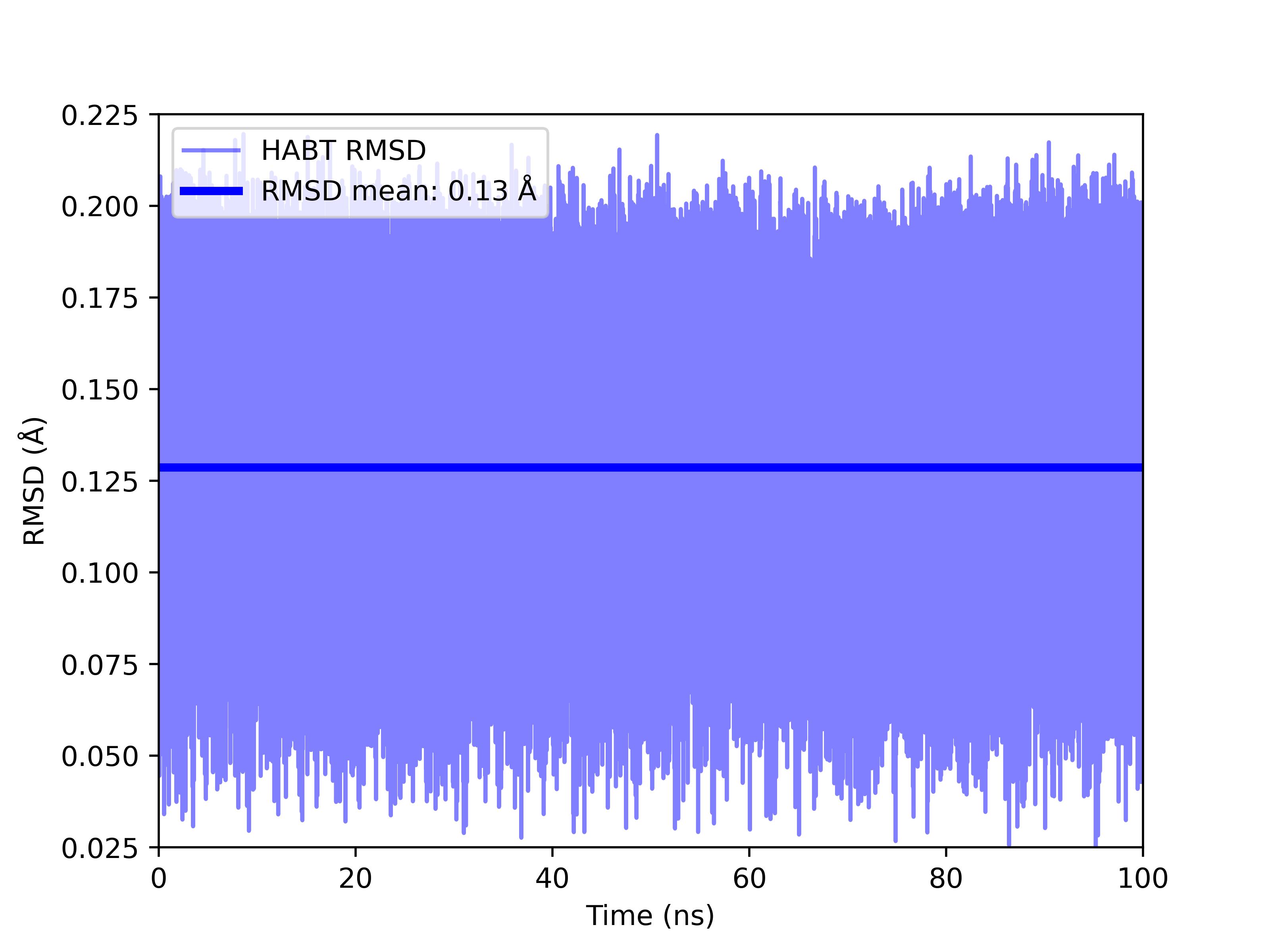

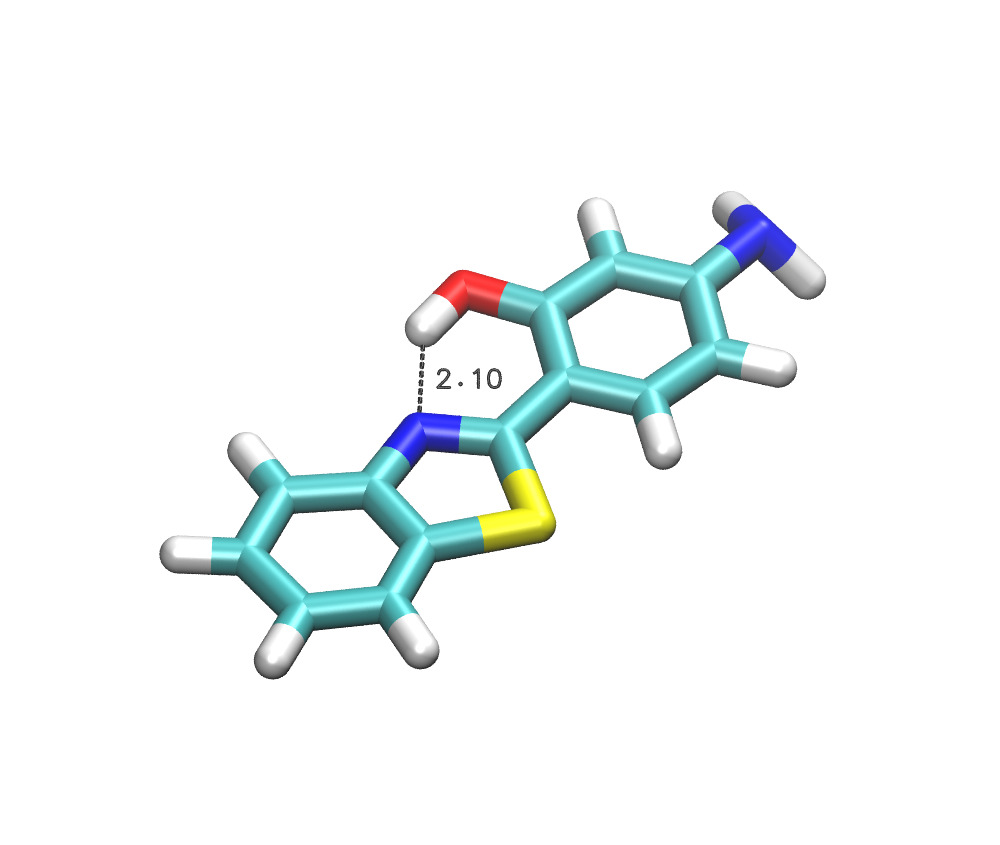


   a) b)


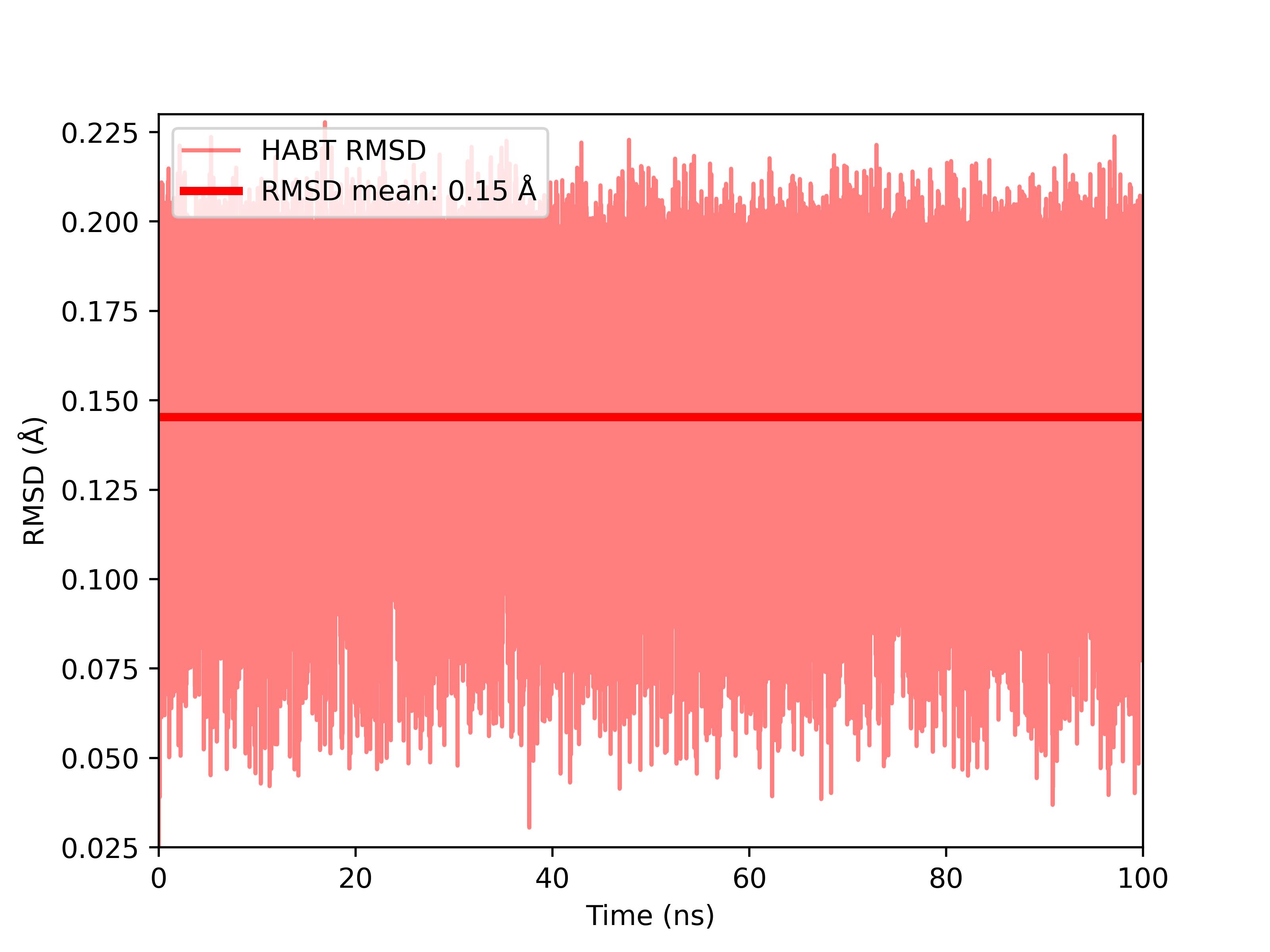

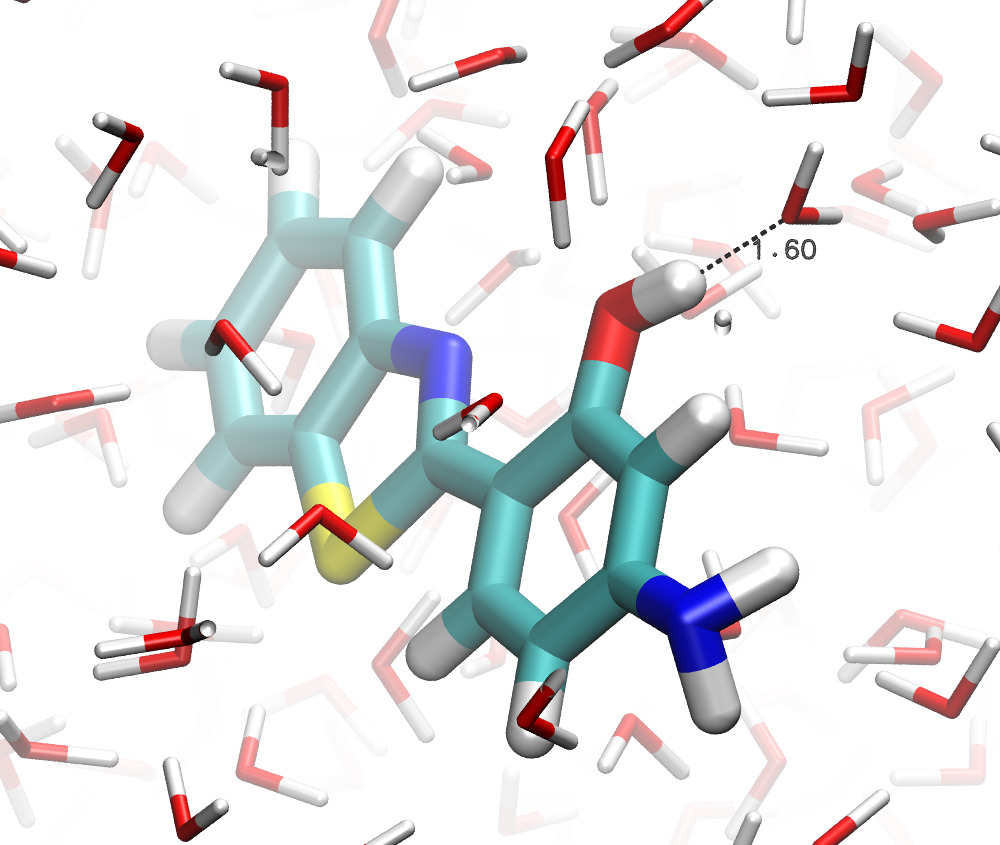


   c) d)

   Figure S1. a) RMSD profile for system 1. b) Intramolecular N···HO h-bond interaction important for system 1 RMSD profile. c) RMSD profile for system 2. d) Intermolecular h-bond between -OH group of HABT and water molecules important for system 2 RMSD profile.

   Since system 1 was simulated in vacuum, only intramolecular interactions can occur. In that scenario, by observing the HABT structure, the only h-bond interaction that can occur is the one between the -OH group of its phenyl portion and the -N= group of its benzothiazole portion, shown in Figure S1b. Thus, this interaction was held for almost all simulated frames of system 1.

   However, after the addition of water, intermolecular h-bond must be considered. In this scenario, despite for system 2 the -OH and -N= intramolecular h-bond interaction still occurs with great relevance, competitive intermolecular h-bond can occur with water molecules. Therefore, it was verified that for system 2 an intermolecular h-bond between the hydrogen of -OH group of HABT and oxygen of water molecules can takes place of the intramolecular h-bond interaction (see Figure S1d). Nevertheless, only a slight increase in its RMSD average was observed for system 2 when compared to system 1. Thus, it was possible to imply that the -OH an -N= intramolecular h-bond interaction kept its great relevance.

   **SI.2.2 Protein Fluctuation**

   An RMSF analysis was performed in system 3 (PROT+HABT+WAT) to evaluate the residues fluctuation throughout the simulation production. From this analysis, additional information about protein stability (discussed in the main text, Section 3.2) could be obtained, observing which residues differentiates the most when compared to its minimized structure. In this sense, Figure S2 shows the obtained RMSF for each amino acid residue. The residues that presented the highest RMSF values are: HIS1089, LYS1000, ILE244, GLN148, LEU245, GLN246, and SER147, with values varying between 0.5369 Å to 0.6792 Å. In the other hand, for the RMSF values of the residues pointed out as important residues to stabilize HABT dihedral 1 and 2 fluctuations, it was obtained for LYS833 an RMSF of 0.1383 Å, and for ASP950 an RMSF of 0.1804 Å, a considerably smaller value, implying that the PI3K cavity responsible to interact with the HABT molecule is similar to the PI3K cavity of the minimized structure.


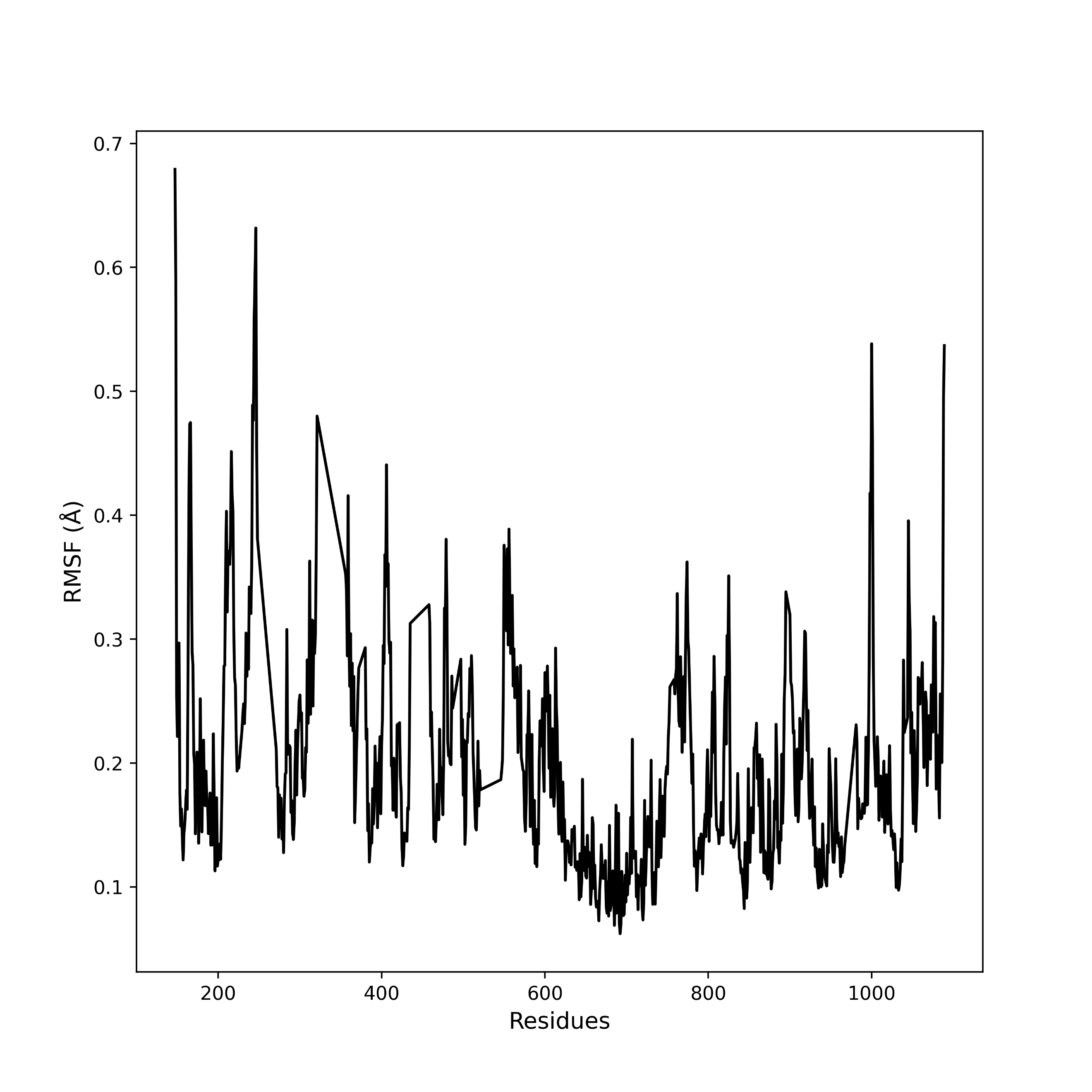


   Figure S2. Obtained RMSF values for each amino acid residue from the Unbiased MD trajectory.

   **SI.3 Biased MD simulation stability**

   In order to evaluate the stability of the system 3 biased MD simulation, an RMSD analysis was conduct, and the result is reported in Figure S3. The RMSD analysis showed an increase in the observed standard deviation, which was of 1.06 Å.


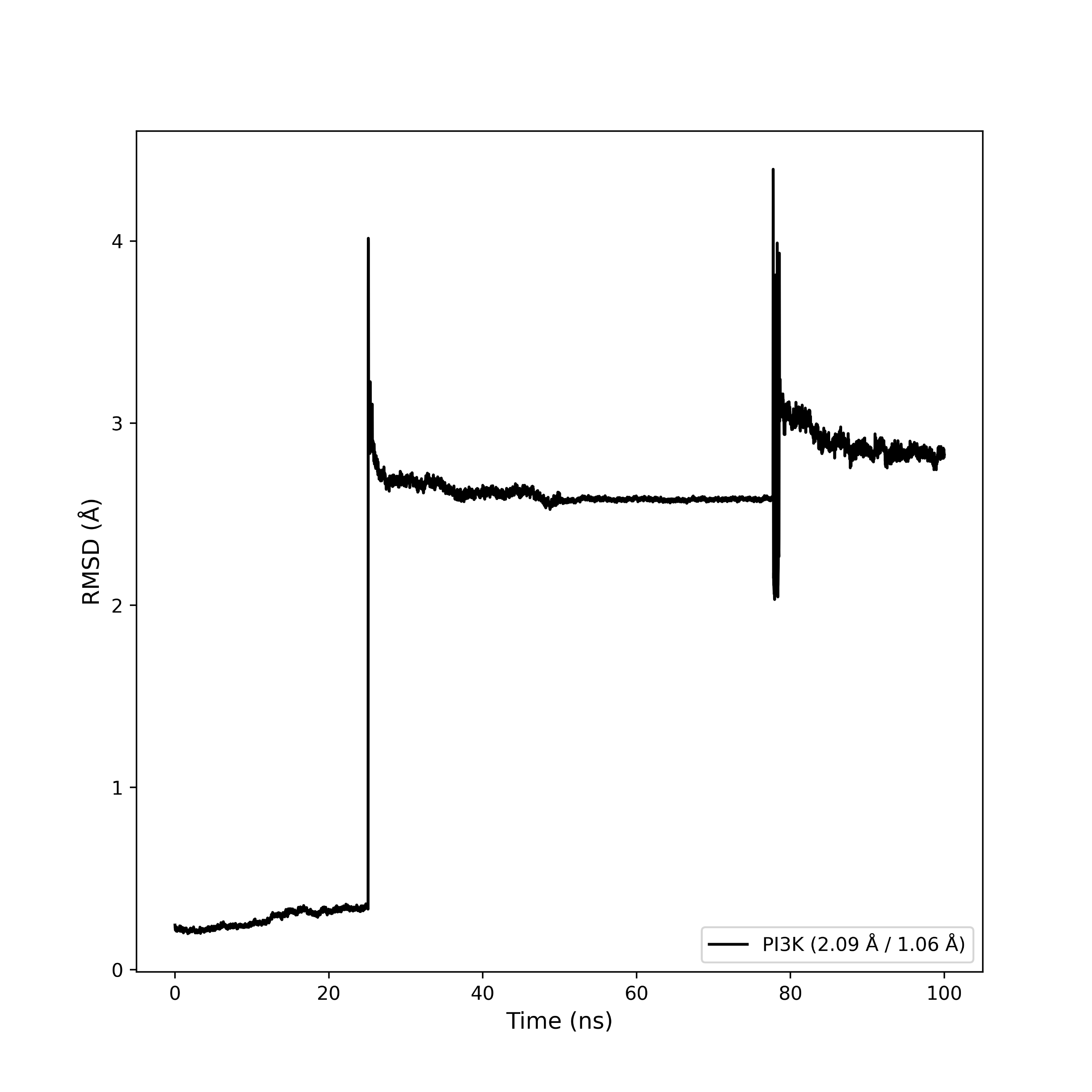


   Figure S3. RMSD analysis conducted for system 3 biased MD simulation trajectory.

   In addition, an RMSF analysis was conducted for system 3 biased MD simulation trajectory to evaluate which residues differed the most from the PI3K minimized structure, and the result is reported in Figure S4. It was observed that the amino acid residues that presented the highest RMSF values were: PHE832, SER824, ILE828, LEU823, THR827, GLU826, and ASN825, with values that range between 2.7385 Å to 2.8729 Å. Now, observing the RMSF values of the residues pointed out as important to stabilized HABT dihedral 1 and 2 fluctuations, for LYS833 an RMSF of 2.5002 Å was observed, and for ASP950 an RMSF of 1.2181 Å was obtained.


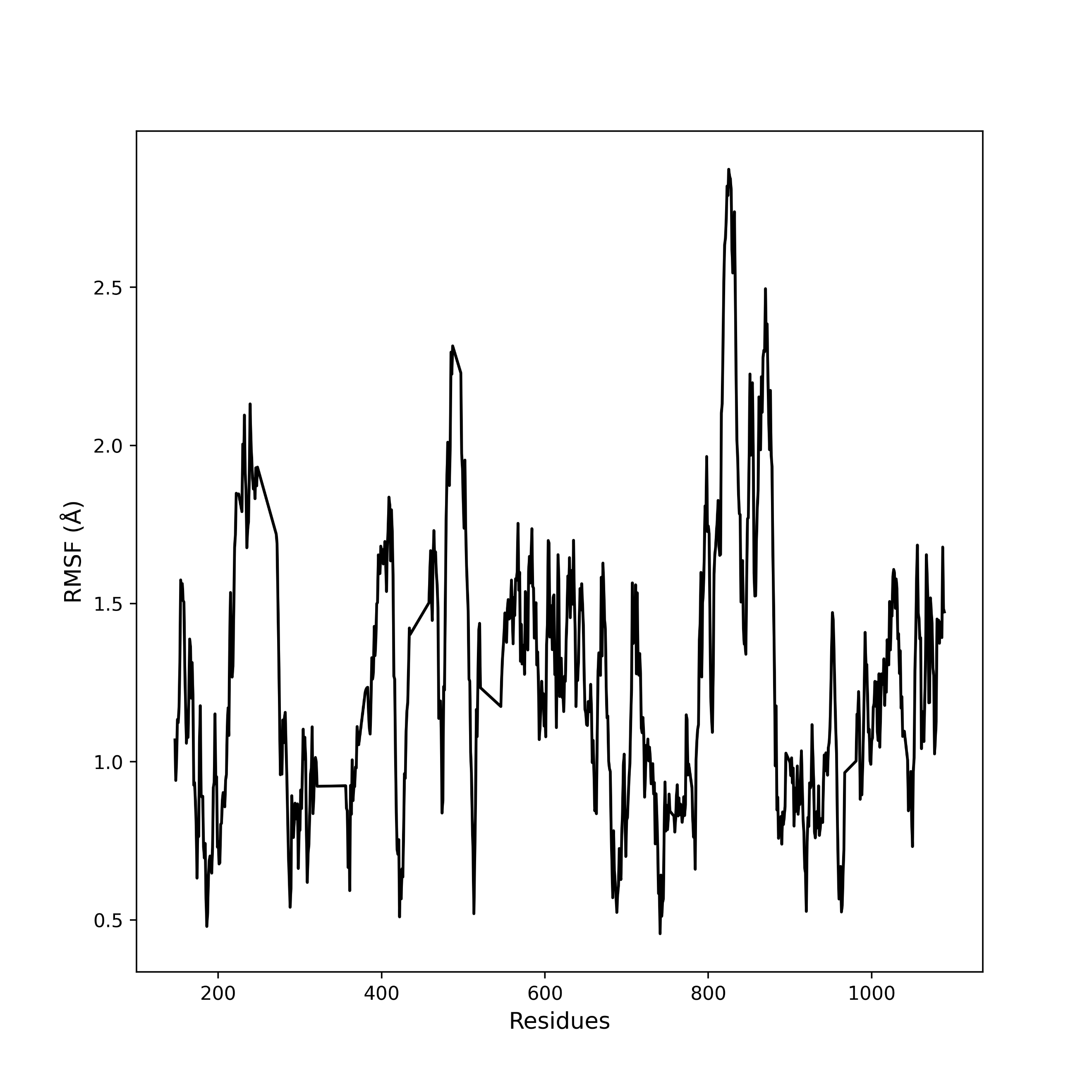


   Figure S4. Obtained RMSF values for each amino acid residue from the Biased MD trajectory.

   It is important to notice an overall increase in RMSF values, as well as an RMSD standard deviation, which implies a higher deviation of the PI3K minimized structure in the biased MD simulation. In that regarded, it is important to mention that the work main goal is to investigate ligand flexibility by accessing its metastable conformations and evaluating its stabilization in the protein environment, where this evaluation was made in terms of dihedrals 1 and 2 of the HABT molecule. For this, the enhanced sampling technique OPES expanded was used to increase ligand flexibility and sample conformations that could not be sampled in relevant populations with only a 100 ns unbiased MD simulation production. Therefore, the increase in RMSD standard deviation and RMSF values are expected, once by biasing the potential energy *U*, not only ligand, but protein fluctuations are also enhanced. However, it is important to mention that the RMSF value of the ASP950 is considerably lower than the ones observed for the most deviated amino acid residues, and since this amino acid residue is the most important in stabilizing the HABT dihedral 2 fluctuation, which is of major importance in ESIPT appropriate geometry evaluation, it shows the reliability of the statistical data provided in the work. Nevertheless, the authors warn that the absolute values in terms of ESIPT spectroscopic emission should be taken with care and encourage new research for a more accurate evaluation in absolute energy terms.

   **SI.4 Metastable states labeling in system 3**


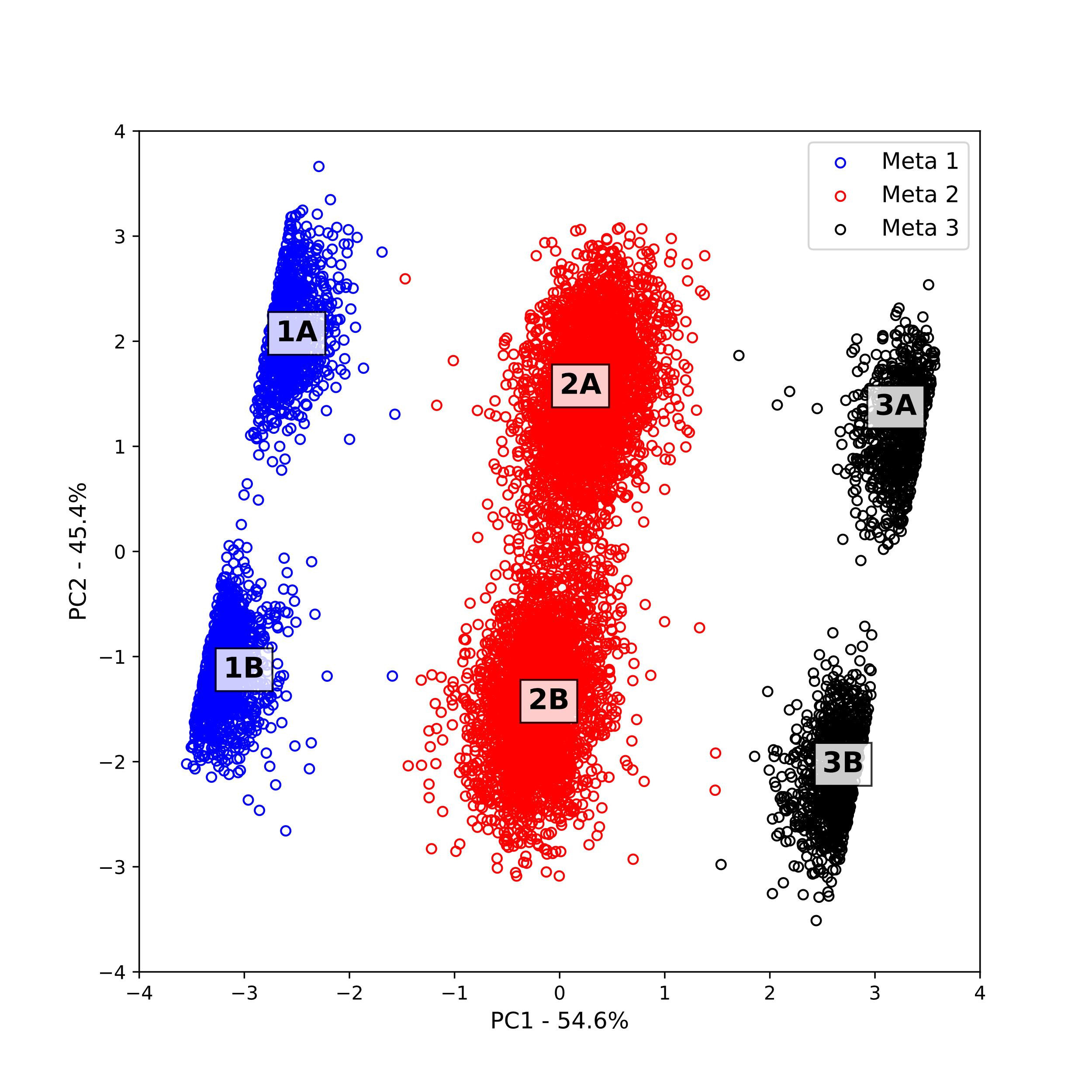


   Figure S5. Labels attached to each observed accessed metastable state in system 3.

   **SI.5 Conformational selection**

   Once identified and characterized the accessed metastable states of system 3, it was possible to move forward and select representative conformations of these metastates, shown in Table S2, for QM calculations. In this scenario, all accessed metastates, long or shorter-lived, were treated in accordance with the previously mentioned labeling, presented in Figure S5. Now, about the conformational selection, the criteria were based on the average values of dihedral 1 and 2.

   In each metastate, the average of dihedral 1 and 2 were calculated, and the conformation whose dihedral 1 and 2 values were the closest of the averages, was selected. In addition to that, the equilibrium conformation was also selected using the same criteria. However, the dihedrals 1 and 2 were calculated from the unbiased trajectory. In this scenario, the average value was computed from all the 10000 frames of the unbiased trajectory. Then, the conformation with the closest dihedral 1 and 2 values from its average was selected. All these values are shown in Table S1.

   In this scenario, Table S2 was constructed in order to show the values of dihedral 1 and 2 of all selected conformations, including a third variable, being it the distance between the group -N= and the hydrogen of the group -OH of HABT molecule. The mentioned distance is of great importance. Since both groups are involved in the ESIPT reaction, it is expected a hampered ESIPT for those conformations whose distance N···HO are larger. The selected conformations for each metastate can be visualized in Table S2.

   It is important to mention that amino acids residues were collected in order to consider the protein environment in QM calculations. In this sense, the amino acid residues ASP950 and LYS833 were collected with the HABT structure in each selected frame. This decision was made in accordance with the performed RMSD and hbond analysis, where both residues were responsible for the observed stabilization of the simulation evolution.

   Therefore, as a general description, it was possible to see that conformations of (3)Meta 2 are the most similar to the equilibrium one, shown in Table S2. On the other hand, conformations of (3)Meta 1 and 3 are the most different of the equilibrium one, presenting fluctuations on both dihedrals, and a higher N···HO distance. For the equilibrium conformation, as expected, no relevant fluctuation was verified for dihedral 1 and dihedral 2, being the closest of the optimized structure.

   | Table S1. Calculated averages for dihedral 1 and 2 in each accessed metastable state. Also, the table presents the average values of distance N···HO in these metastable states. | | | |
   | --- | --- | --- | --- |
   | Metastable State | Dihedral 1 (rad) | Dihedral 2 (rad) | Distance N···HO (Å) |
   | 1A | 1.53 | -2.92 | 4.46 |
   | 1B | -1.75 | -2.91 | 4.58 |
   | 2A | 1.50 | -0.03 | 2.95 |
   | 2B | -1.47 | 0.04 | 2.93 |
   | 3A | 1.75 | 2.91 | 4.59 |
   | 3B | -1.61 | 2.93 | 4.49 |
   | *EQ* | *0.07* | *-0.16* | *3.75* |

   | Table S2. Table shows the values of dihedral 1, 2 and distance N···HO of each representative conformation selected. | | | | |
   | --- | --- | --- | --- | --- |
   | Conformation | Visualization | Dihedral 1 (rad) | Dihedral 2 (rad) | Distance N···HO (Å) |
   | 1A | 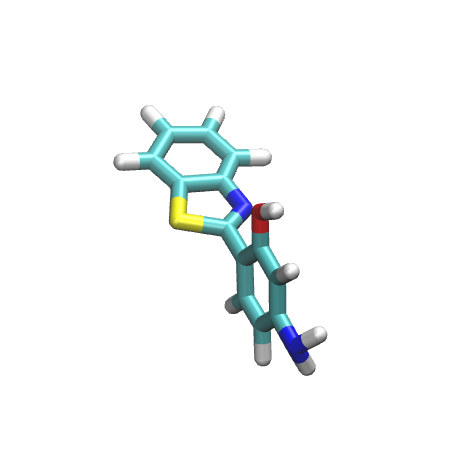 | 1.52 | -2.90 | 4.40 |
   | 1B | 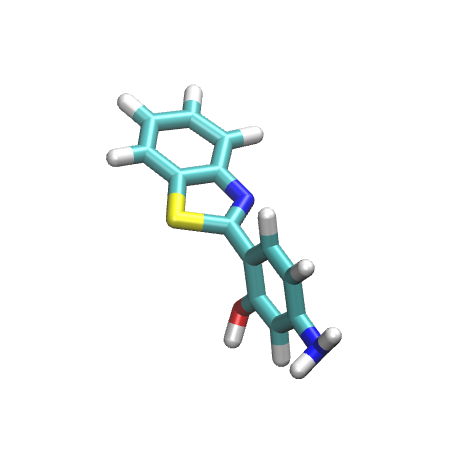 | -1.74 | -2.91 | 4.49 |
   | 2A | 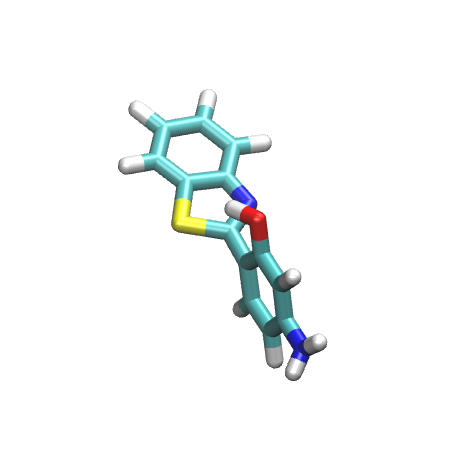 | 1.49 | -0.04 | 2.94 |
   | 2B | 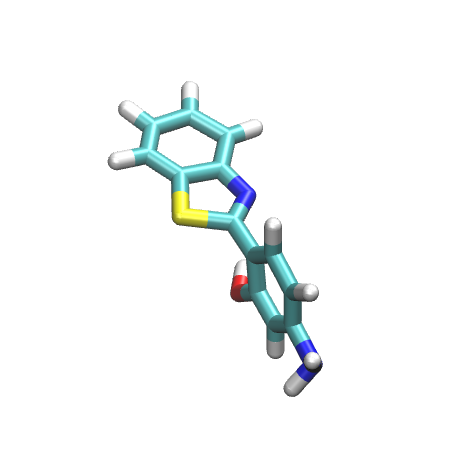 | -1.46 | 0.02 | 2.61 |
   | 3A | 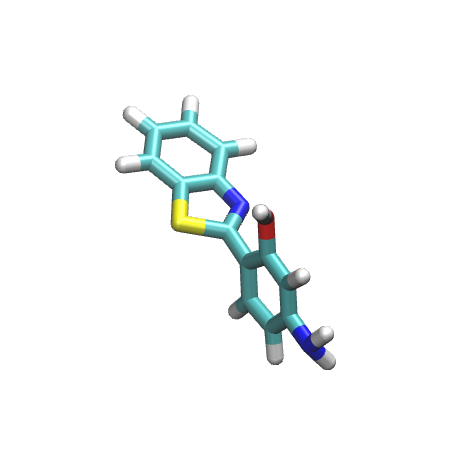 | 1.72 | 2.91 | 4.53 |
   | 3B | 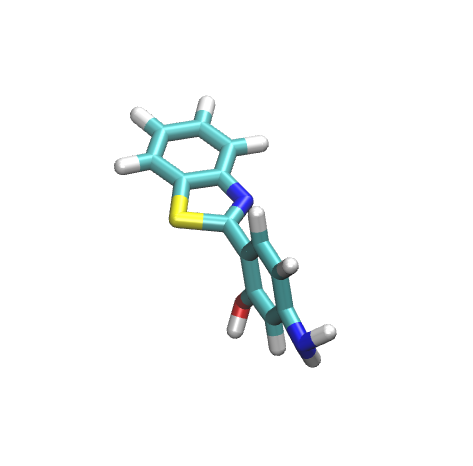 | -1.58 | 2.92 | 4.48 |
   | *EQ* | *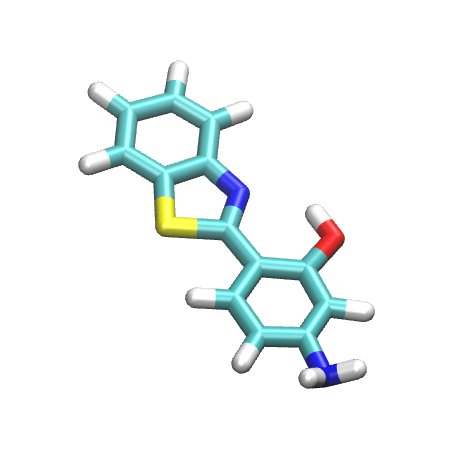* | *0.03* | *-0.16* | *2.30* |

   **SI.6 QM/MM calculation - ONIOM Setup**

   QM/MM calculations were performed to compare the data with the obtained results in the spectroscopy section of the main text. However, due to the computational cost, the QM/MM calculations were performed only for the Conformations 2B, 3A, and *EQ* (See Table S2). For such calculation, Gaussian 09 software was used, were for input setup GaussView 6.0 was used in order to define the ONIOM layers (QM and MM region). Figure S6 shows how the regions were defined, where in red (HABT, ASP950, and LYS833) is the QM region, and in blue (rest of the protein) the MM region. The QM region was treated with the B3LYP functional and TZVP basis set. On the other hand, MM region was treated using the Universal Forcefield (UFF). First, HABT geometry was optimized in each state described in main text Section 2.6, maintaining the amino acids residues LYS833 and ASP950 fixed with the rest of the protein. With the optimized HABT geometries, single point calculations were performed to investigate the vertical excitation energies.

   **
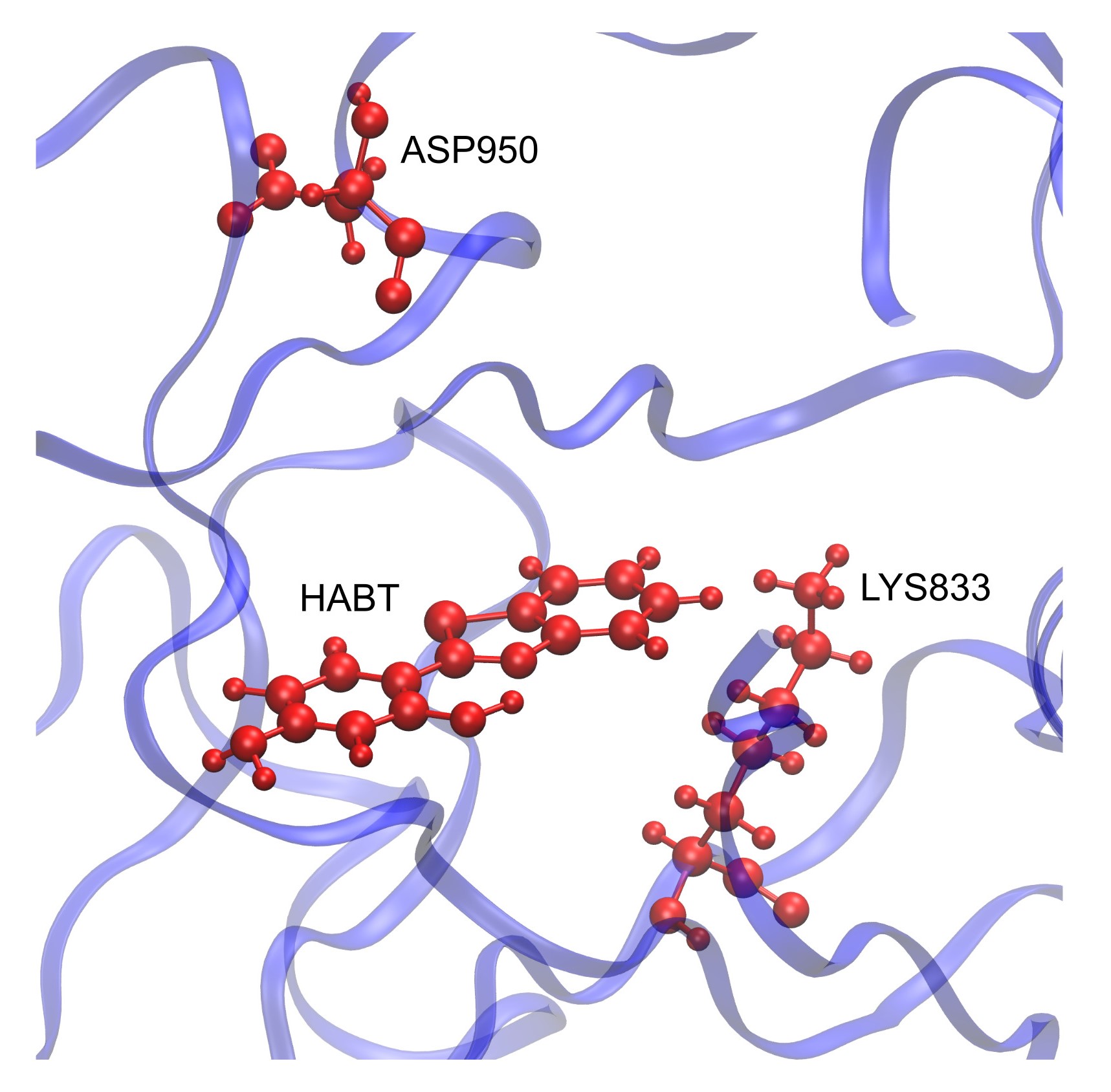
**

   Figure S6. Defined QM and MM regions for the QM/MM calculation. In red, the QM region considers the HABT molecule and the amino acid residues aspartic acid (ASP950) and lysine (LYS833). In blue, the MM region considers all the rest of the protein.

   Table S3 shows the obtained enol and keto excitation energies for the conformations 2B, 3A, and *EQ*. It was possible to notice a decrease in the enol excitation energies for all conformations, otherwise, for the keto emission, conformation 2B presented a small increase, and *EQ* conformation a small decrease. The results showed that, when considering the protein in the MM region, the enol emission gets closer to the UV-VIS region, and the keto emission maintain in the blue/green region.

   | Table S3. Calculated excitation energies for the conformations with QM/MM method. ΔE1 accounts for the enol excitation energy, and ΔE3 for the keto excitation energy. | | |
   | --- | --- | --- |
   | Conformation | ΔE1 | ΔE3 |
   | 1A | - | - |
   | 1B | - | - |
   | 2A | - | - |
   | 2B | 3.66 | 2.73 |
   | 3A | 3.22 | - |
   | 3B | - | - |
   | *EQ* | *3.10* | *2.32* |

   **SI.7 Dihedral Scan – Comparison between QM and MM**

   A dihedral scan was performed for both QM and MM levels of theory to observe the behavior of GROMOS 54a7 forcefield and compare it with the QM behavior. The scanned dihedral corresponds to dihedral 1 (shown in main text). The QM scan was made in Gaussian 09 software, where the energies were calculated for a range of dihedrals lying between 0 and 360 degrees, with a step size of 10 degrees. For that, B3LYP functional was used with TZVP basis set. Now, the MM dihedral scan was also performed for a range of dihedrals lying between 0 and 360 degrees, with a step size of 10 degrees, using GROMOS 54a7 forcefield and GROMACS 2024.1 software.

   The dihedral 1 QM and MM scan shown to be similar in profile. Figure S7 shows the obtained profile for both scans, where it was possible to observe that, both scans presented their lowest energies in the 0 and 360 degrees approximately, values where an intramolecular h-bond interaction can occur between -OH and -N= group (See Figure S1b). The observed result agrees with the observed from the unbiased MD production, where the equilibrium conformation (see Table S2), which is the most populated conformation, presents a dihedral 1 with negligible fluctuation. Another important result is that, both scans showed peaks in energy around 90, 180, and 270 degrees. In this sense, GROMOS 54a7 ff showed a similar profile when compared with the QM scan.


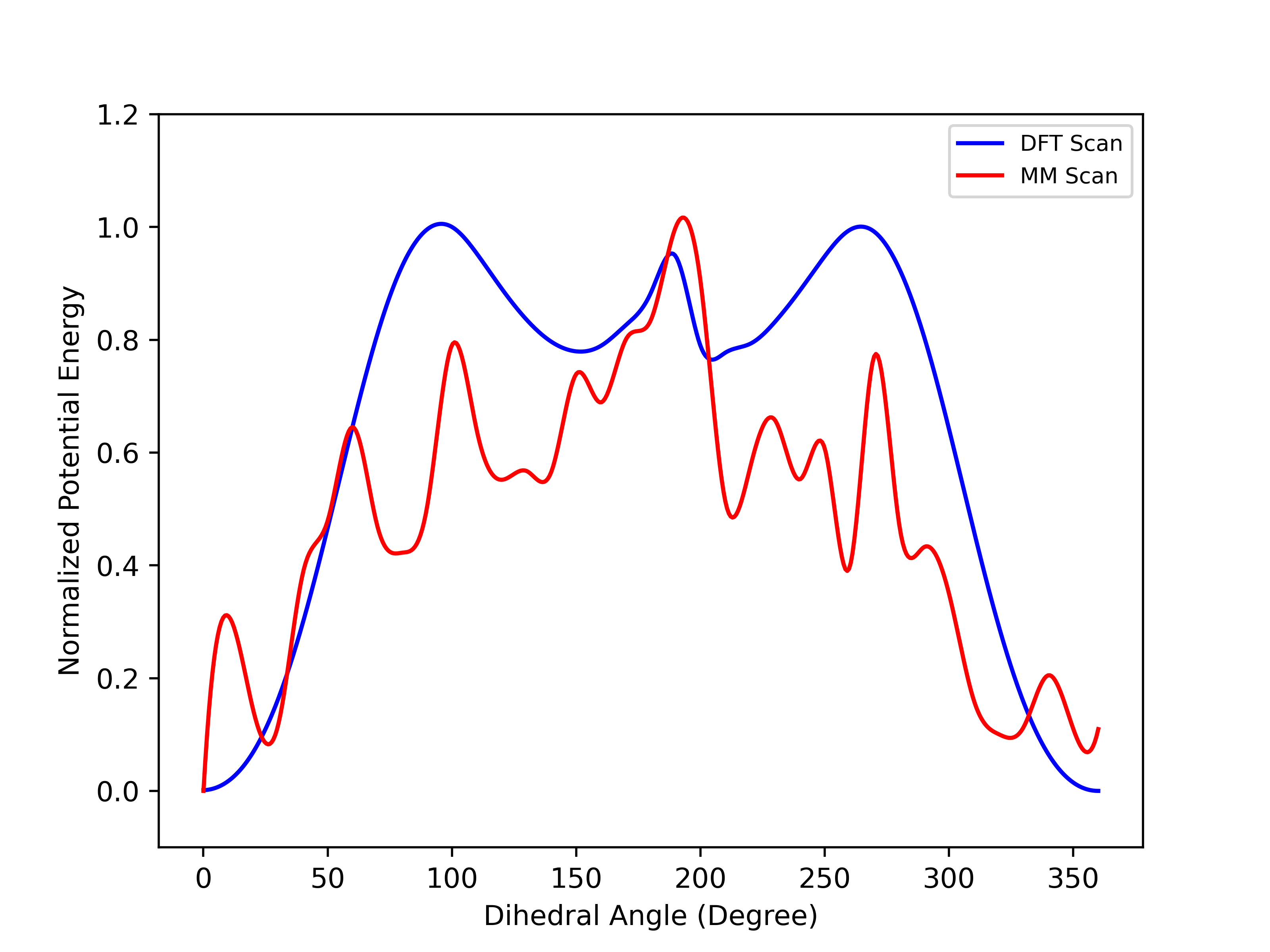


   a)


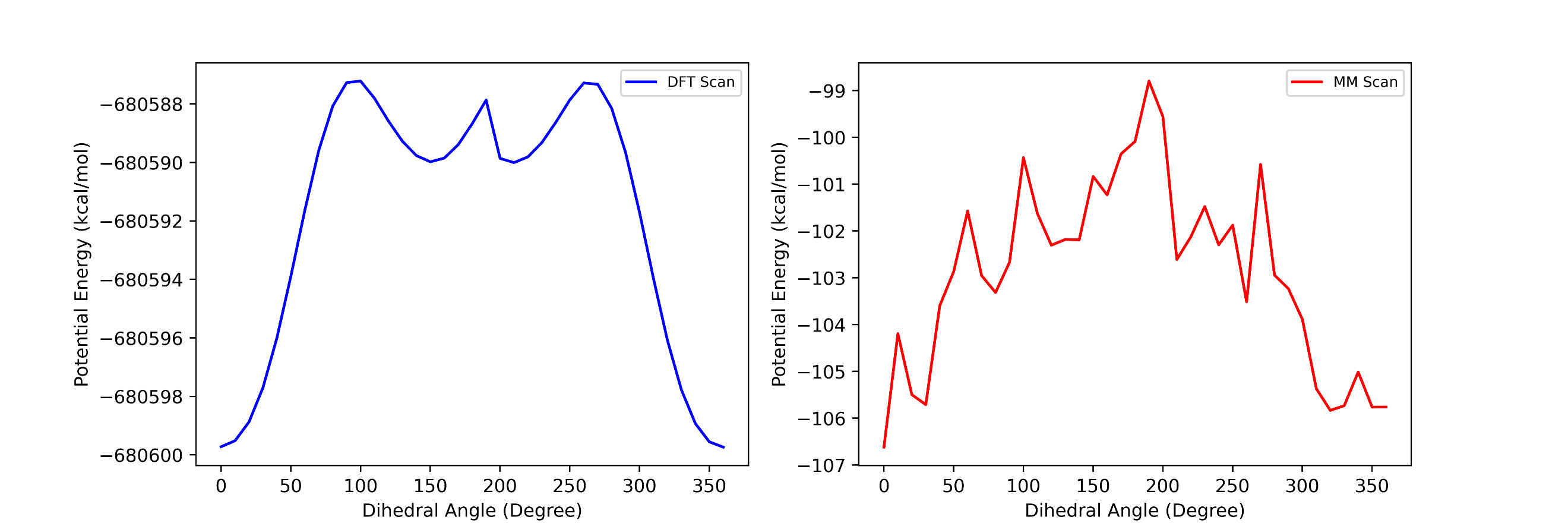


   b) c)

   Figure S7. In blue, QM dihedral 1 scan using B3LYP functional and TZVP basis set, and in red, MM dihedral 1 scan using GROMOS 54a7 forcefield. To make a relative comparison between the obtained profiles, a) presents the potential energies normalized, and b) and c) presents the non-normalized potential energies.

   In addition, the energy barriers associated with dihedral 1 transitions were calculated in both QM and MM scan, the computed values are reported in Table S4. In total, three energy barriers were observed in the presented profiles in Figure S7, being them: the energy barrier associated with the first peak, around 90º (E1), the energy barrier associated with the second peak, around 180º (E2), and the energy barrier associated with the third peak, around 270º (E3). The obtained values showed a good agreement between QM and MM scans, where the MM energy barriers presented a small increase when compared to the QM energy barriers.

   | Table S4. Calculated energy barriers for both QM and MM dihedral 1 scan. The reported values are shown in kcal/mol. | | |
   | --- | --- | --- |
   | Energy Barrier | QM scan | MM scan |
   | E1 | 2.629 | 2.882 |
   | E2 | 2.151 | 2.433 |
   | E3 | 2.868 | 2.935 |

   [↑](#endnote-ref-2)
